# RACK1 Associates with Muscarinic Receptors and Regulates M_2_ Receptor Trafficking

**DOI:** 10.1371/journal.pone.0013517

**Published:** 2010-10-20

**Authors:** Cindy L. Reiner, Jennifer S. McCullar, Rebecca L. Kow, Joshua H. Le, David R. Goodlett, Neil M. Nathanson

**Affiliations:** 1 Department of Pharmacology, University of Washington, Seattle, Washington, United States of America; 2 Institute for Systems Biology, Seattle, Washington, United States of America; Iowa State University, United States of America

## Abstract

Receptor internalization from the cell surface occurs through several mechanisms. Some of these mechanisms, such as clathrin coated pits, are well understood. The M_2_ muscarinic acetylcholine receptor undergoes internalization via a poorly-defined clathrin-independent mechanism. We used isotope coded affinity tagging and mass spectrometry to identify the scaffolding protein, receptor for activated C kinase (RACK1) as a protein enriched in M_2_-immunoprecipitates from M_2_-expressing cells over those of non-M_2_ expressing cells. Treatment of cells with the agonist carbachol disrupted the interaction of RACK1 with M_2_. We further found that RACK1 overexpression inhibits the internalization and subsequent down regulation of the M_2_ receptor in a receptor subtype-specific manner. Decreased RACK1 expression increases the rate of agonist internalization of the M_2_ receptor, but decreases the extent of subsequent down-regulation. These results suggest that RACK1 may both interfere with agonist-induced sequestration and be required for subsequent targeting of internalized M_2_ receptors to the degradative pathway.

## Introduction

Endocytosis of cell surface proteins has several functions, e.g., delivery of nutrients and viruses into the cell. Internalization of cell surface receptors allows for coupling to additional signal transduction pathways [1], resensitization and recycling to the cell surface for further signaling [2], or degradation (down regulation) [3]. Endocytosis can occur both constitutively, as in the case of the transferrin receptor, or in response to agonist stimulation, as in the case of most G-protein coupled receptors (GPCRs).

Agonist-induced receptor internalization can be characterized by the receptor's dependence on clathrin coated pits. Clathrin coated pit endocytosis involves the assembly of a clathrin matrix and is the most common form of endocytosis for GPCRs [4]. One clathrin independent pathway is caveolar internalization, which involves caveolin and is associated with lipid rafts [5], [6]. However, not all receptors internalize through these two pathways. For instance, the M_2_ muscarinic acetylcholine receptor (mAChR) does not associate with clathrin or caveolin in JEG-3 and HEK cells [7]–[12].

Recent developments in the field of proteomics have allowed the identification of the components of protein complexes. Specifically, isotope-coded affinity tagging (ICAT) of proteins allows the comparison of the composition of two protein complexes by labeling them separately with either a heavy or light isotope [13]. The relative level of individual proteins in each sample can then be quantified. We employed this method with the intent of identifying proteins associated with the M_2_ receptor following agonist stimulation. In this study, we compared protein complexes immunoprecipitated from cells transfected with the M_2_ receptor to those from mock transfected cells. We identify RACK1 as a protein that interacts with the M_2_ muscarinic receptor in an agonist-regulated manner and that selectively regulates its internalization and downregulation.

## Results

In order to identify proteins that interact with the M_2_ receptor, we first established a protocol that could isolate the putative M_2_ internalization complex. We chose the JEG-3 cell line for these experiments because our lab has previously demonstrated that M_2_ internalizes through a relatively uncharacterized pathway in these cells [10], [12]. Because the protein-protein interactions involved in endocytosis may be transient or disrupted by detergents, we used a cleavable membrane-permeable crosslinker to covalently link proteins coming into contact with M_2_ following agonist stimulation. Using a protocol adapted from Min et al. [14], we were able to immunoprecipitate a high molecular weight complex from JEG-3 cells transfected with Flag-M_2_ and stimulated with carbachol (data not shown).

To specifically identify polypeptides associated with the M_2_ receptor, we used ICAT, a technique in which the protein content of two samples is compared by covalently labeling peptides from the sample and control preparations with a heavy or light isotope [13]. When these samples are combined and analyzed by mass spectrometry the difference in molecular weight between the heavy and light isotopes allows their origin to be tracked and area under the curves for their respective single ion current traces allows quantification of relative protein ratios between the two samples. We used an ICAT modification, called solid-phase isotope labeling, in which the isotopes are conjugated to beads [15]. This modification retains more protein sample in the wash steps and is thus better suited for experiments where isolating large amounts of protein is impractical.

Immunoprecipitates from mock transfected cells were used as a control. Protein samples from multiple transfections were pooled for an estimated total of 1 µg M_2_ protein. We compared the Flag-M_2_ and mock-transfected protein complexes by capturing cysteine-containing peptides with beads conjugated to light (d0) or heavy (d6) isotopes, respectively. The samples were then combined and washed and the labeled peptides were cleaved from the beads and analyzed by MS/MS.

Despite the small amount of protein, we identified at least one protein of interest. Receptor for activated protein kinase C (RACK1), identified by a single peptide (FSPNSSNPIIVSCGWDK) with a probability score of 1, was 2 fold more abundant in the M_2_ containing samples. RACK1 is a member of the tryptophan-aspartate (WD) repeat family and is a scaffolding protein with many known binding partners and functions [16].

**Figure 1:**
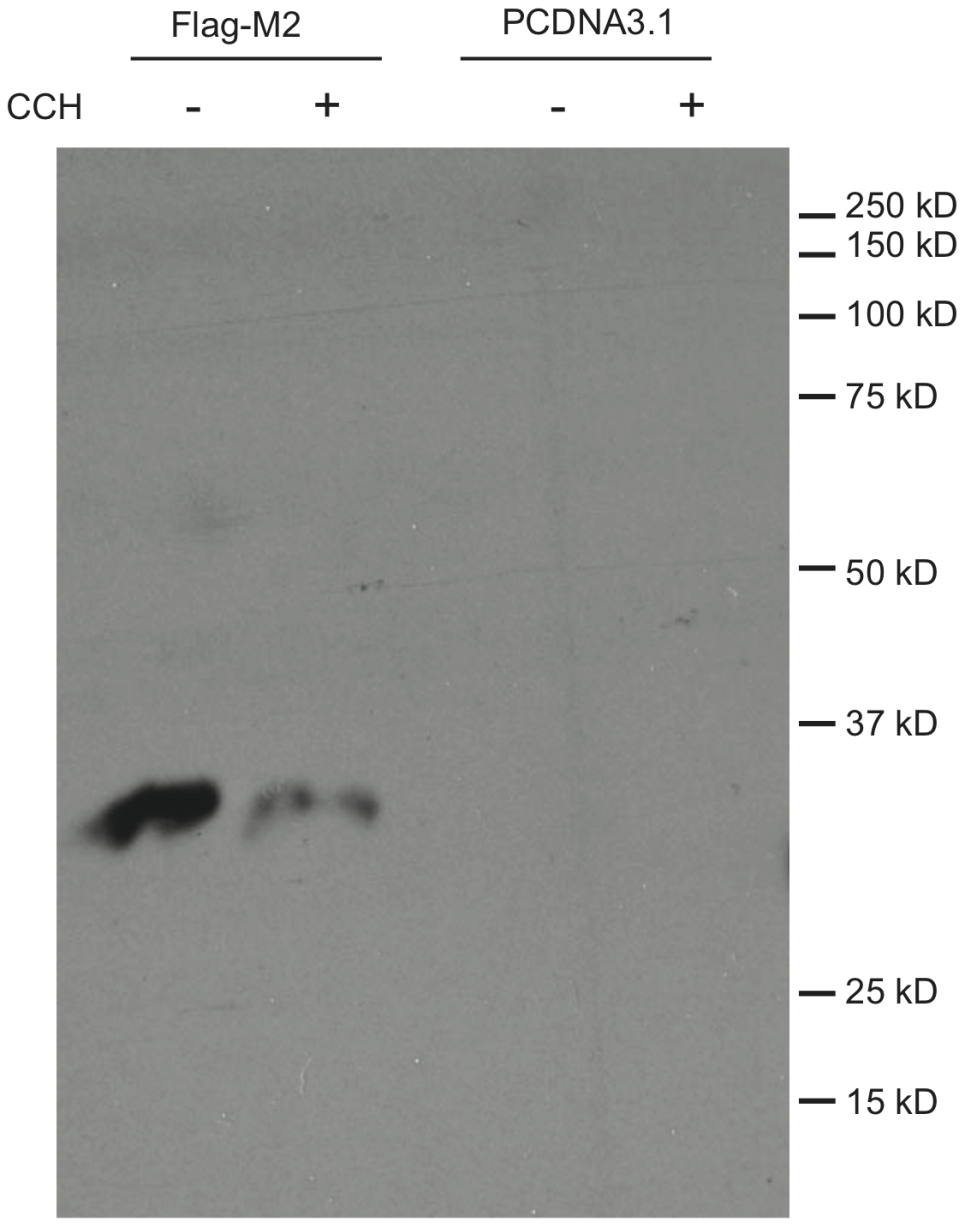
Agonist-sensitive interaction of RACK1 with M_2_. HEK cells stably expressing either PCDNA3.1 or Flag-M_2_ were treated with 1 mM carbachol (CCH) for 30 minutes as indicated. Receptors were immunoprecipitated with anti-Flag antibodies and run on SDS-PAGE. Anti-RACK1 antibodies were used to blot. Blot shown is representative of ≥3 experiments.

In order to confirm an interaction between M_2_ and RACK1, we used immunoprecipitation of Flag-M_2_ from stably transfected HEK cells with anti-Flag antibodies, followed by Western blot analysis with anti-RACK1 antibodies. We also tested the effects of carbachol stimulation on the interaction by immunoprecipitation from unstimulated and carbachol-stimulated cells, and compared the amount of RACK1 immunoprecipitated by anti-Flag antibody from non-M_2_ expressing cells as a test for the specificity of co-immunoprecipitation (Fig. 1). We found that RACK1 and M_2_ specifically co-immunoprecipitate and that this interaction is disrupted by carbachol treatment, despite having used agonist stimulated cells in the original proteomics experiments. We also found that RACK1 could be co-immunoprecipitated with M_2_ in an agonist-sensitive manner from transiently transfected JEG-3 cells (data not shown.) Interestingly, the crosslinker used in the proteomics experiment was not necessary to observe the interaction between RACK1 and M_2_ in either cell type.

**Figure 2:**
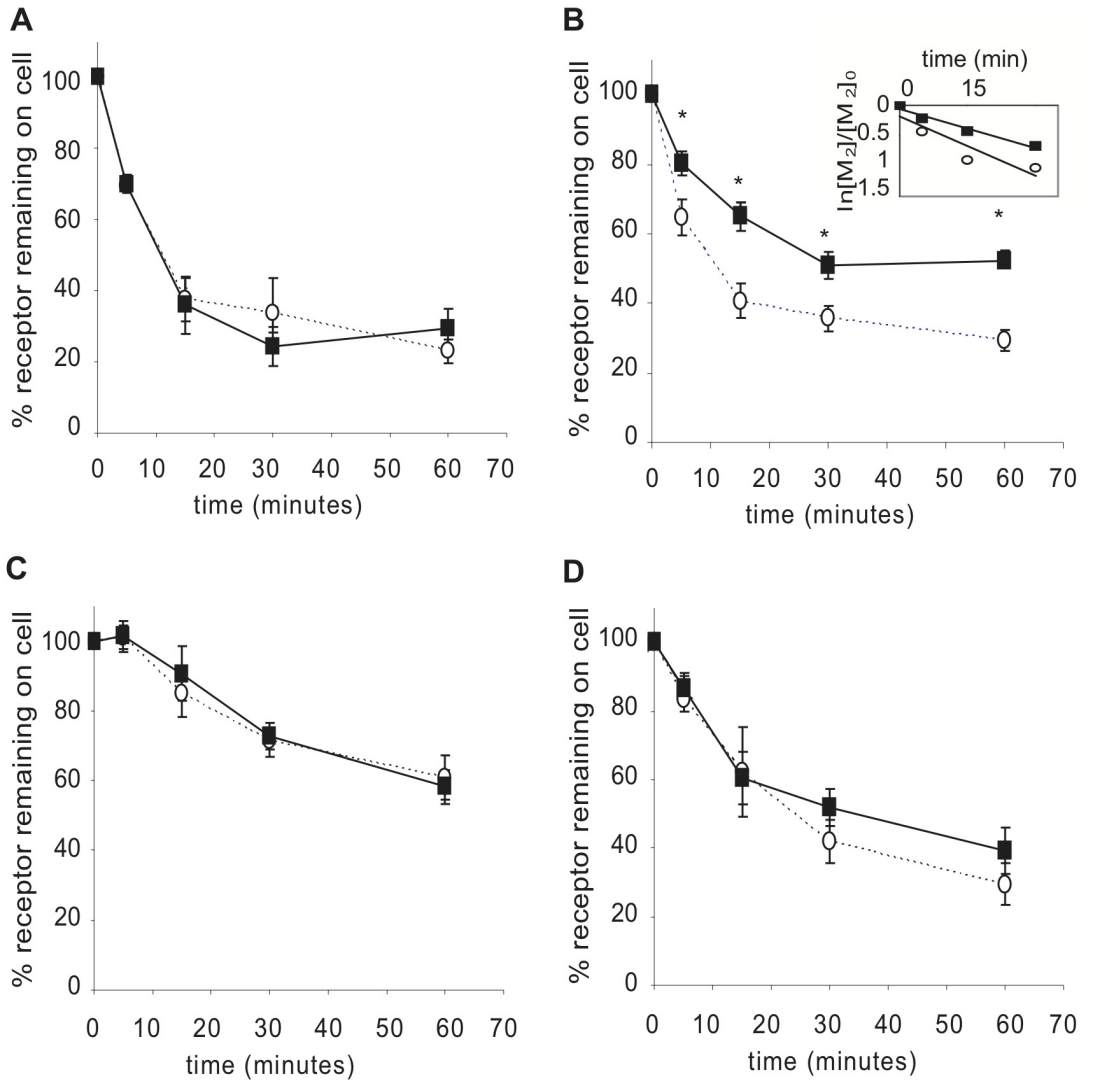
Effects of RACK1 overexpression on mAChR internalization. HEK cells were cotransfected with either M_1_ (A), M_2_ (B), M_3_ (C), or M_4_ (D) and either PCDNA3.1 (open circles) or RACK1 (black squares). Cell surface receptors were measured by [^3^H]NMS binding following stimulation for 5, 15, 30 and 60 minutes with 1 mM carbachol. Internalization is expressed as percent of receptor remaining on the cell surface compared to control unstimulated cells. *Inset*: data from panel B were fit to an exponential decay curve. The rate-constants are M_2_ + PCDNA3.1, 3.2×10^−2^ min^−1^; M_2_ + RACK1, 2.1×10^−2^ min^−1^. *indicates p≤0.04.

RACK1 has been implicated in the trafficking of several GPCRs to the membrane [17], [18]. We tested the effects of over expression of RACK1 in transiently expressing HEK cells by co-transfecting Flag-M_2_ with either PCDNA3.1 or RACK1 into HEK cells and used the membrane-impermeable antagonist ([^3^H]NMS) binding to measure cell surface receptors after 5, 15, 30 and 60 minutes of carbachol stimulation (Fig. 2B). We found similar receptor expression levels at time 0 and a significant decrease in both the rate and extent of M_2_ internalization when RACK1 was overexpressed, with only about 40% of the receptors internalized after 60 minutes compared to almost 70% of receptors internalized in cells overexpressing M_2_ only. To determine if this effect was specific to the M_2_ receptor, we tested whether RACK1 overexpression would also affect M_1_, M_3_, and M_4_ internalization (Fig. 2A, 2C, and 2D). There was not a significant change in the internalization of any of these receptors when RACK1 was over expressed. Thus, over expression of RACK1 regulates the internalization of the M_2_ mAChR in a receptor subtype-specific fashion.

**Figure 3:**
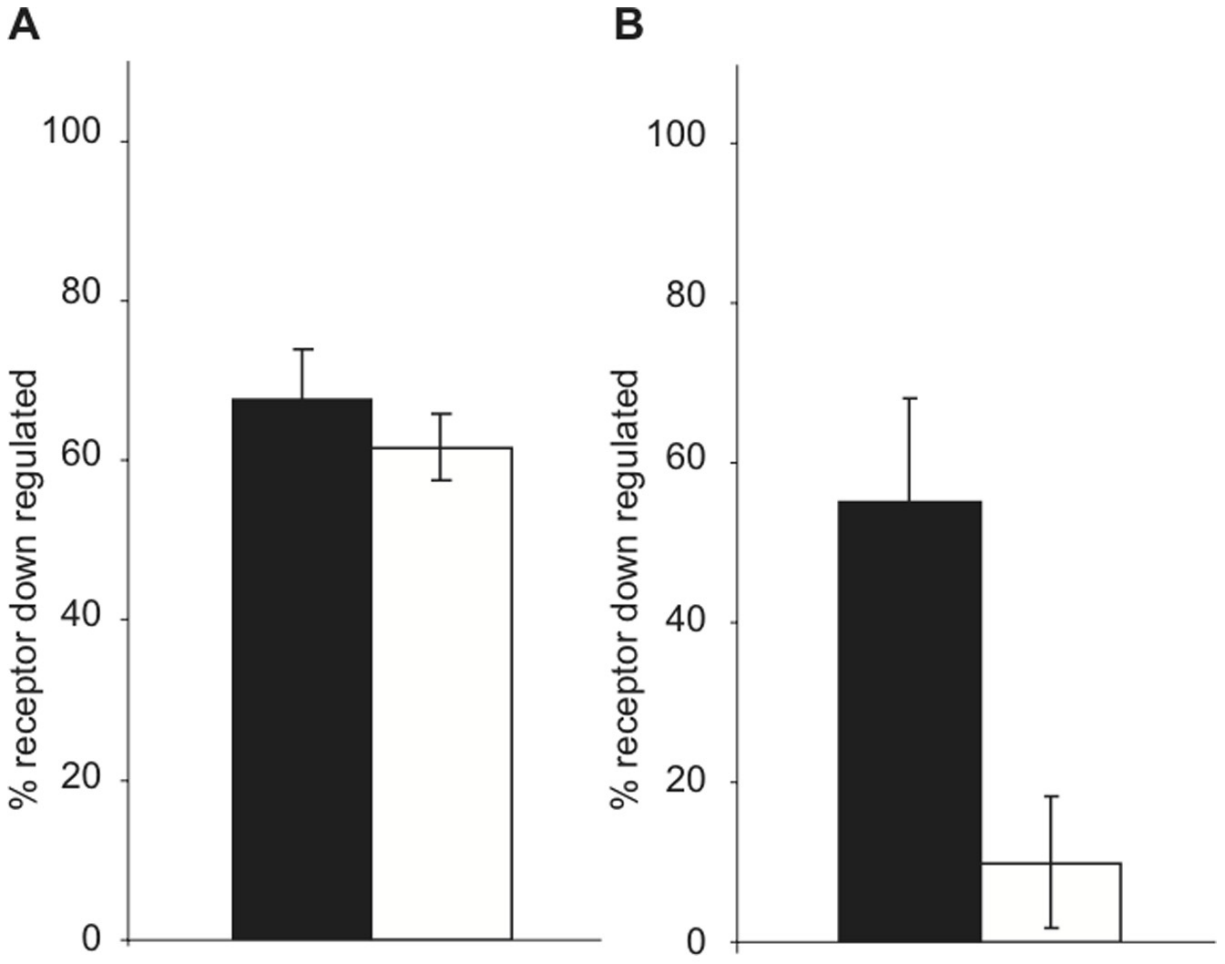
Effects of RACK1 overexpression on mAChR down regulation. HEK cells were cotransfected with either M_1_ (A) or M_2_ (B) and either PCDNA3.1 (black bars) or RACK1 (white bars). Total receptors were measured by [^3^H]QNB binding following 8 hours of stimulation with 1 mM carbachol. Down regulation is expressed as percent of control cells. * indicates p<0.001.

Following internalization, receptors are either recycled back to the cell surface or targeted to the lysosome for degradation (down regulated). Because M_2_ is not recycled in HEK cells [19], we next examined the effects of RACK1 on M_1_ and M_2_ receptor down regulation. Cells cotransfected with receptor and either PCDNA3.1 or RACK1 were treated with 1 mM carbachol for 8 hours and total receptor was measured by labeling with the membrane permeable radioligand [^3^H]QNB. We found that while the cell types had similar expression levels at time zero, RACK1 severely inhibited M_2_ down regulation with only around 10% of receptors being down regulated compared to over 50% of receptors down regulated in cells transfected with M_2_ alone (Fig. 3B), but had no effect on M_1_ down regulation (Fig. 3A).

**Figure 4:**
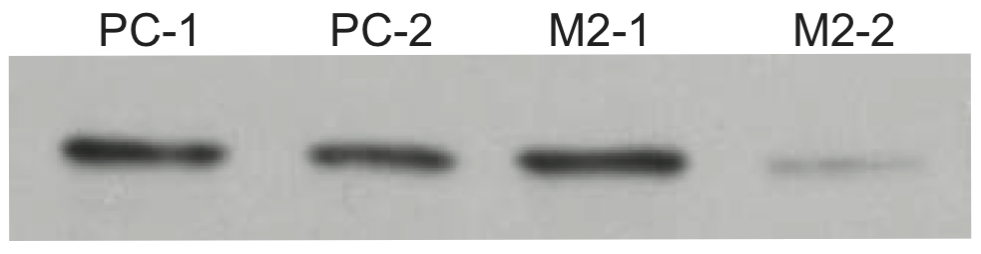
RACK1 in lysates of HEK cells stably expressing M_2_. Lysates from four clonal HEK cell lines stably transfected with either PCDNA3.1 (PC-1 and PC-2) or Flag-M_2_ (M2-1 and M2-2) were run on SDS-PAGE. Anti-RACK1 antibodies were used to blot. Blot shown are representative of ≥3 experiments.

**Figure 5:**
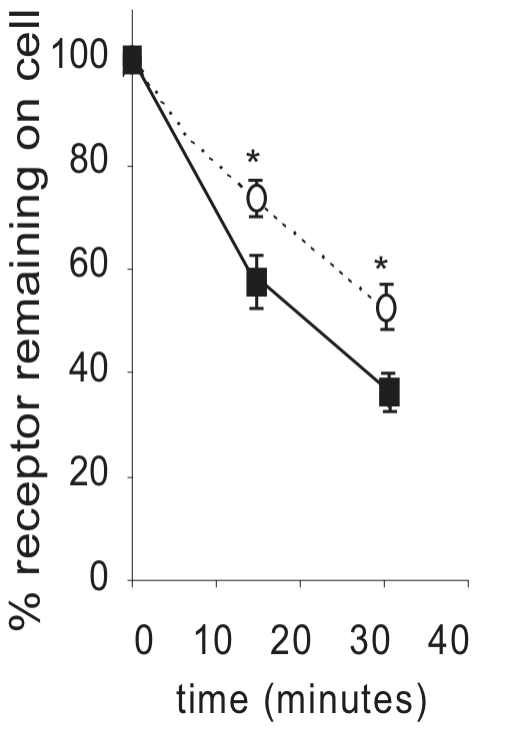
Effects of decreased RACK1 expression on M_2_ mAChR internalization. HEK cells stably expressing Flag-M_2_ with normal levels of RACK1 (cell line M2-1; open circles) or low levels of RACK1 (cell line M2-2; black squares) expression. Cell surface receptors were measured by [^3^H]NMS binding following stimulation for 15 or 30 minutes with 1 mM carbachol. Internalization is expressed as percent of receptors remaining on the cell surface compared to unstimulated cells. * indicates p<0.02.

**Figure 6:**
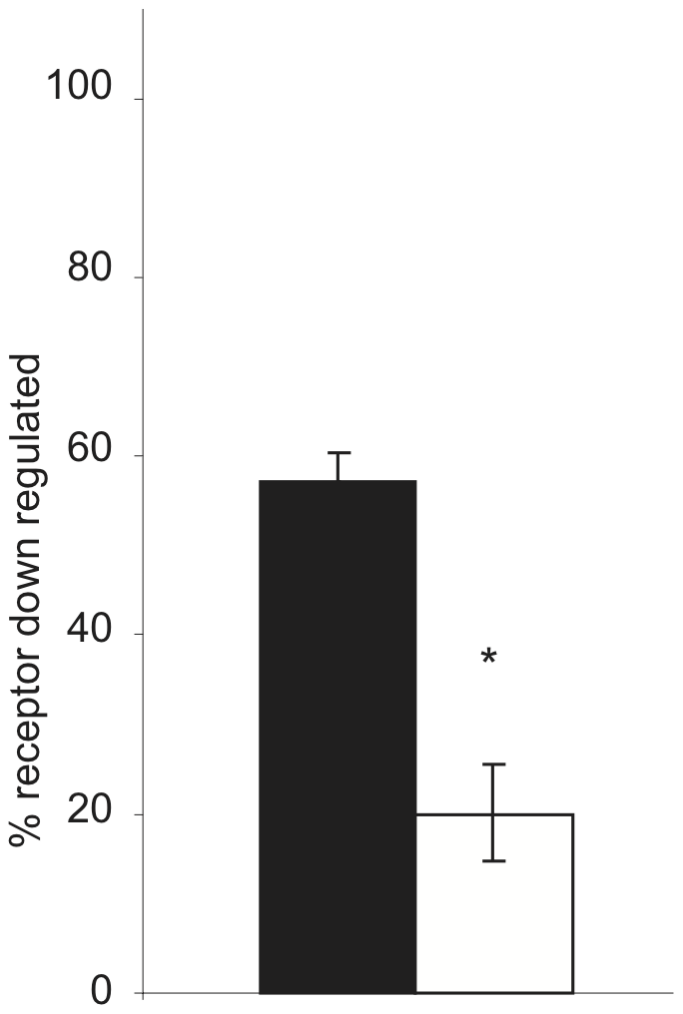
Effects of decreased RACK1 expression on M_2_ mAChR down regulation. HEK cells stably expressing Flag-M_2_ with either normal levels of RACK1 (black bar) or with low levels of RACK1 (white bar) expression. Total receptors were measured by [^3^H]QNB binding following 8 hours of stimulation with 1 mM carbachol. Down regulation is expressed as percent of receptor down regulated compared to unstimulated cells. * indicates p<0.001.

During the course of isolating stably transfected M_2_ expressing cells, we found that one M_2_ expressing clonal cell line (M2-2) expressed significantly lower levels of RACK1 compared both to several clonal lines transfected with pCDNA3.1 alone (PC-1, PC-2) and to another M_2_ expressing clonal cell line (M2-1), with M2-2 expressing approximately 1/5 the level of the M2-1 cell line as determined by densitometry analysis (Fig. 4). While RACK1 could be co-immunoprecipitated with M_2_ in M2-1 cells (Fig. 1), we were not able to detect RACK1 co-immunoprecipitation from the M2-2 cells (data not shown). We took advantage of this to test the effects of the decreased levels of RACK1 expression on M_2_ trafficking. Receptor expression in both cell lines was found to be mainly (≥80%) on the cell surface in unstimulated conditions. We found that in cells with decreased RACK1 expression levels there is an increase in the extent of M_2_ internalization with over 60% of receptors internalized after 30 minutes compared to almost 45% of receptors internalized in cells with normal levels of RACK1 expression (Fig. 5). When we tested M_2_ receptor down regulation in the stable cell lines relatively lacking in RACK1, we found that down regulation was again inhibited with only about 20% of receptors down regulated compared to almost 60% down regulated in cells with normal levels of RACK1 expression (Fig. 6).

## Discussion

In this study, we report that RACK1 interacts with M_2_ and regulates trafficking of the receptor following agonist stimulation. This interaction was first identified using solid phase isotope labeling and mass spectrometry and confirmed by co-immunoprecipitation and Western blot analysis. Although the initial assay was designed to identify proteins that associate with agonist-stimulated M_2_, the co-immunoprecipitation and Western blot analysis found that RACK1 dissociates from M_2_ following agonist stimulation.

We tested the effects of both overexpression of RACK1 as well as decreased expression of RACK1 on M_2_ trafficking following agonist stimulation. The internalization of M_2_, which occurs within an hour of carbachol stimulation, is inhibited when RACK1 is over expressed and enhanced when RACK1 is lacking. Taken together with the disassociation of RACK1 from M_2_ following stimulation, it is likely that the interaction of RACK1 with M_2_ prevents the receptor from undergoing endocytosis. It has been suggested that the interaction of M_2_ with beta-arrestin following agonist stimulation is required for receptor internalization [20]. Beta-arrestin and RACK1 have overlapping binding sites on phosphodiesterase 4D5 and decreased levels of RACK1 lead to an increase in beta-arrestin binding to PDE4D5 [21]. It is possible that a similar competition is taking place on the M_2_ receptor with low levels of RACK1 allowing increased beta-arrestin binding and increased internalization, while high levels of RACK1 prevent beta-arrestin from acting on the M_2_ receptor. Additionally, RACK1 over expression could be acting as a regulator of receptor phosphorylation. M_2_ phosphorylation has been shown to be sufficient for receptor internalization [22]. In a similar fashion, RACK1 associates with the NMDA receptor where it prevents phosphorylation until the interaction is disrupted by stimulation [23]. We further found that RACK1 specifically regulates the internalization of the M_2_ receptor, as RACK1 over expression did not affect the internalization of any of the other mAChR subtypes. This is not surprising given that the M_2_ receptor has been shown to internalize through a novel, yet relatively uncharacterized, pathway [7]–[12].

The role of RACK1 in M_2_ down regulation is more complex as both increased and decreased levels of RACK1 inhibit M_2_ down regulation. It is likely that there are differing causes for this seeming disparity. The inhibition of M_2_ down regulation due to excess RACK1 is most easily explained by RACK1 inhibition of M_2_ internalization. If the receptors remain on the cell surface, they will not be targeted to the lysosome for degradation. The inhibition of M_2_ down regulation in the cells with decreased levels of RACK1 could be due to the inability of internalized vesicles to reach the lysosome. It is possible that RACK1 is involved in targeting the endosomes to the lysosomes in a similar fashion to its role in targeting recycling vesicles to the centrosome during mitosis in C. elegans [24].

In addition to its effects on M_2_ receptor trafficking, RACK1 could regulate receptor function in other ways. Our finding that RACK1 dissociates from M_2_ following receptor activation suggests that RACK1 may regulate M_2_ signal transduction. There are many possible pathways that could be affected by this interaction, including regulation of GIRK channels [25] and signaling through PKC [26] or through the beta-gamma subunit of heterotrimeric G-proteins [27] or acetylcholinesterase [16]. Furthermore, the stimulation dependence of the RACK1/mAChR interaction is similar to that of the NMDA receptor, where RACK1 acts as an inhibitory scaffolding protein, preventing NMDA receptor phosphorylation until the proteins dissociate following stimulation [28]. This raises the possibility that RACK1 could be either a positive or negative regulator of mAChR signaling.

In summary, we have identified a novel interaction between RACK1 and the M_2_ mAChR, where RACK1 specifically regulates M_2_ internalization and down regulation. RACK1 has multiple protein binding partners and affects a variety of cellular functions. Further investigation into the association of RACK1 with mAChRs is necessary to fully understand the importance of RACK1 in mAChR signaling.

## Materials and Methods

### Materials

Dulbecco's modified Eagle's medium (DMEM), fetal bovine serum (FBS) and penicillin-streptomycin (P/S) were obtained from Life Technologies. Lipofectamine2000 was from Invitrogen. N-[^3^H]methylscopolamine ([^3^H]NMS, 80–82 Ci/mmol), [^3^H]quinuclidinyl benzilate ([^3^H]QNB, 49 Ci/mmol), and ECL reagents were purchased from Amersham. The anti-IgM antibody, and dithiobis(succinimidylpropionate) (DSP) were from Pierce. The anti-RACK IgM antibody was from BD Biosciences. The Centricon filter devices and Immobilon-P were from Millipore. The anti-Flag mouse monoclonal M2 antibody, 3× Flag peptide, carbamylcholine chloride (carbachol), atropine, pepstatin, leupeptin, phenylmethylsulfonyl fluoride and all other reagents were purchased from Sigma.

### Plasmids

The Flag-M_2_ pcDNA3.1 construct was generated by digesting pCDPS-Flag-M_2_ [10] with Kpn1 and EcoR1 to remove the Flag-M_2_ coding region, which was then ligated into the pCDNA3.1 vector (Invitrogen). The RACK1 expression vector was the generous gift of Dr. Chris Cartwright (Stanford University) [29].

### Cell Culture and Transfection

JEG-3 choriocarcinoma cells and human embryonic kidney (HEK) cells (American Type Culture Collection, Rockville, MD) were grown in DMEM supplemented with 10% FBS and 1% P/S in a 10% CO_2_ environment at 37° C. For assays, a 15 cm plate was transected using either Lipofectamine2000 or the calcium phosphate method [30] with 25 µg DNA each of both receptor and RACK1 or empty expression vectors, and processed as described below.

For isolation of stably transfected HEK cell lines, cells at 70–80% confluency on 15 cm plates were transfected using the calcium phosphate method with 50 µg of either pCDNA3.1 or the RACK1 expression vector. One day later, cells were split 1∶15 into fresh medium, and on the next day medium containing 750 µg/ml G418 was added. Individual colonies were picked and assayed for mAChR expression.

### Crosslinking and Immunoprecipitation of M_2_ receptor-containing complex

The crosslinking and immunoprecipitation procedure was adapted from previously published methods [14]. At 48 hours post transfection 15 cm plates of JEG-3 cells were washed three times with Buffer A (0.15 M NaCl, 20 mM HEPES, pH 7.4) and stimulated with 1 mM carbachol in 7.5 ml Buffer A. Five minutes following stimulation, 500 µl of freshly made 25 mM dithiobis(succinimidylpropionate) (DSP) in dimethyl sulfoxide was added and plates were returned to the 37° C incubator for 25 minutes. Cells were placed on ice and rinsed with ice cold DMEM, then incubated with DMEM for 5 minutes. After rinsing with buffer A, cells were incubated with lysis buffer (1% nonidet P-40, 4 mg/ml dodecyl-β-D-maltoside, 0.8 mg/ml cholesteryl hemisuccinate in buffer A) plus protease inhibitors for one hour on ice. Cells were removed from the plates and sonicated. After centrifugation, the supernatant was immunoprecipitated with anti-Flag antibody overnight at 4° C. Beads were washed 4× with lysis buffer, then proteins were eluted on a column by incubation with 0.1 mg/ml 3× flag peptide 3 times for 30 minutes. Eluent was concentrated in centricon tubes and resuspended in 200 mM Tris (pH 8.0). Samples from 50 transfected 15 cm plates, and 50 untransfected control plates, were used for solid-phase isotope labeling.

### Solid-phase Isotope Labeling

Protein complexes were labeled and identified using a protocol developed at the Institute for Systems Biology [15]. Samples were digested with 2 µg trypsin (Promega, sequencing grade modified, 1∶20 w/w) overnight at 37° C, then reduced with 2 µl of 250 mM Tris(2-carboxyethyl)phosphine for 30 minutes. Reduced samples were added in the dark to UV-cleavable isotope conjugated beads (d0/d6-gamma-aminobutyric acid beads [15]). Samples collected from mock transfected cells labeled with the light (d0) isotope and those collected from Flag-M_2_ transfected cells labeled with the heavy (d6) isotope. After shaking 15 minutes, the labeling reaction was quenched with 100 µl water and 2 µl beta-mercaptoethanol. d0 and d6 beads were combined and loaded onto a Bio-Spin column then washed with 1 ml each three times with 1.5 M NaCl, 6 times with 0.1% trifluoroacetic acid, 6 times with 80% N-phenylethanamide, 0.1% trifluoroacetic acid, 3 times with MeOH, 3 times with 1∶9 NH_4_OH:MeOH, 6 times with MeOH and 6 times with water. The beads were resuspended in 100 µl 20 mM Tris, pH 8 and 2 µl beta-mercaptoethanol. Proteins were cleaved from the beads by exposure to UV light for 2 hours and then washed from the beads 3 times with 100 µl 80% N-phenylethanamide, 0.1% trifluoroacetic acid. The sample was concentrated by Speed-Vac and resuspended in 5 µl 0.2% acetic acid [15]. Peptides were analyzed by µHPLC-MS and data dependent µHPLC-MS/MS acquisition, selecting from each survey scan the top-three most abundant precursor ions for collision induced dissociation with a dynamic exclusion of 1 [13]. For this, a linear ion trap mass spectrometer (LTQ; Thermo Finnigan, San Jose, CA) was used with an in-house fabricated microelectrospray source and an HP1100 solvent delivery system (Agilent, Palo Alto, CA). Samples were automatically delivered by a FAMOS autosampler (LC Packings, San Francisco, CA) to a 100 µm internal diameter fused silica capillary pre-column packed with 2 cm of 200 Å pore-size Magic C18AQ™ material (Michrom Bioresources, Auburn, CA) as described elsewhere [31]. SEQUEST™ (Thermo Finnigan) was used to determine peptide sequence and PeptideProphet™ [32] was used to verify correctness of peptide assignments.

### Co-immunoprecipitation and Western Blotting

Stably transfected HEK cells expressing either Flag-M_2_ mAChR or pCDNA3.1 were split onto 15 cm plates and allowed to grow to 90% confluence. Plates were treated for 30 min with or without 1 mM carbachol, then placed on ice and washed with 10 ml ice cold Buffer A. Cells were then resuspended in Buffer A and counted. An equal number of cells from each treatment were then centrifuged at 500× g for 10 min and resuspended in 1 ml cold lysis buffer. Samples were then sonicated, centrifuged and the supernatant was immunoprecipitated with anti-Flag antibodies and protein G agarose beads overnight at 4° C. Beads were washed 6 times with lysis buffer, then resuspended in 4× SDS sample buffer. Samples were spun again and supernatent was run on 12% polyacrylamine gels then transferred to Immobilon-P. The membrane was then immunoblotted with anti-RACK IgM antibody and analysed by ECL.

### Internalization of mAChRs

Cell surface expression of mAChRs was measured using the binding of the membrane impermeable radioligand N-[^3^H]methylscopolamine ([^3^H]NMS) to intact cells using a previously described method [33]. Briefly, 24 hours post-transfection each 15 cm plate was split into three 6-well plates. 48 hours post-transfection, triplicate wells were stimulated with 1 mM carbachol for 0, 5, 15, 30 or 60 min. Triplicate control wells also received 1 µM atropine to measure non-specific binding. All cells were placed on ice and washed three times with ice-cold phosphate-buffered saline (PBS: 4.3 mM Na_2_HPO_4_, 1.4 mM KH_2_PO_4_, 137 mM NaCl, 2.7 mM KCl, pH 7.4). All wells were then incubated for four hours at 4°C with 1 nM [^3^H]NMS in 2 ml PBS. Following the incubation, all wells were washed three times with ice-cold PBS, solublized with 0.5 ml 1% Triton X-100 and transferred to scintillation vials containing 3.5 ml scintillation fluid for counting. The percent of receptor remaining on the cell surface was determined by normalizing the 5, 15, 30 and 60 minute time points to the 0 min time point for the corresponding transfection.

Cell surface expression in stably transfected HEK cells was measured with the same protocol except that 70–80% confluent 15 cm plates were split into two 6 well plates and the binding experiment was carried out the following day.

### Down Regulation of mAChRs

Total cellular expression of mAChRs was measured using the binding of the membrane permeable radioligand [^3^H]quinuclidinyl benzilate ([^3^H]QNB) to intact cells using a previously described method [34]. Briefly, 24 hours post-transfection each 10 cm plate was split into two 6-well plates. 48 hours post-transfection, triplicate wells were stimulated with 1 mM carbachol for 8 hours. All cells were placed on ice and washed three times with ice-cold PBS. All wells were then incubated for 90 minutes at room temperature with 0.6 nM [^3^H]QNB in 2 ml PBS. Three control wells also received 1 µM atropine to measure non-specific binding. Following the incubation, all wells were washed three times with ice-cold PBS and filtered over GF/C filters. The filters were washed 3 times with PBS and transferred to scintillation vials containing 3.5 ml scintillation fluid for counting. The percent of down regulation was determined by normalizing the 8 hour time point to the 0 min time point for each transfection type.

Down regulation of mAChR in stably transfected HEK cells was measured using the same protocol except that 70–80% confluent 15 cm plates were split into two 6 well plates and the binding experiment was carried out the following day.

### Statistical analysis

ANOVA was performed comparing all data from each experiment using Stat View (SAS, Cary, NC) statistical analysis software. For ANOVA with significant *p* values (p<0.05), the Fisher's PLSD post-hoc test was performed to obtain *p* values between control and experimental samples.

## References

[1] Pierce KL, Luttrell LM, Lefkowitz RJ (2001). New mechanisms in heptahelical receptor signaling to mitogen activated protein kinase cascades.. Oncogene.

[2] Claing A, Laporte SA, Caron MG, Lefkowitz RJ (2002). Endocytosis of G protein-coupled receptors: roles of G protein-coupled receptor kinases and beta-arrestin proteins.. Prog Neurobiol.

[3] von Zastrow M (2003). Mechanisms regulating membrane trafficking of G protein-coupled receptors in the endocytic pathway.. Life Sci.

[4] Le Roy C, Wrana JL (2005). Clathrin- and non-clathrin-mediated endocytic regulation of cell signalling.. Nat Rev Mol Cell Biol.

[5] Nichols B (2003). Caveosomes and endocytosis of lipid rafts.. J Cell Sci.

[6] Parton RG, Richards AA (2003). Lipid rafts and caveolae as portals for endocytosis: new insights and common mechanisms.. Traffic.

[7] Delaney KA, Murph MM, Brown LM, Radhakrishna H (2002). Transfer of M2 muscarinic acetylcholine receptors to clathrin-derived early endosomes following clathrin-independent endocytosis.. J Biol Chem.

[8] Roseberry AG, Bunemann M, Elavunkal J, Hosey MM (2001). Agonist-dependent delivery of M(2) muscarinic acetylcholine receptors to the cell surface after pertussis toxin treatment.. Mol Pharmacol.

[9] Roseberry AG, Hosey MM (2001). Internalization of the M2 muscarinic acetylcholine receptor proceeds through an atypical pathway in HEK293 cells that is independent of clathrin and caveolae.. J Cell Sci.

[10] Schlador ML, Nathanson NM (1997). Synergistic regulation of m2 muscarinic acetylcholine receptor desensitization and sequestration by G protein-coupled receptor kinase-2 and beta-arrestin-1.. J Biol Chem.

[11] van Koppen CJ (2001). Multiple pathways for the dynamin-regulated internalization of muscarinic acetylcholine receptors.. Biochem Soc Trans.

[12] Reiner C, Nathanson NM (2008). The internalization of the M2 and M4 muscarinic acetylcholine receptors involves distinct subsets of small G-proteins.. Life Sci.

[13] Gygi SP, Rist B, Gerber SA, Turecek F, Gelb MH (1999). Quantitative analysis of complex protein mixtures using isotope-coded affinity tags.. Nat Biotechnol.

[14] Min L, Galet C, Ascoli M (2002). The association of arrestin-3 with the human lutropin/choriogonadotropin receptor depends mostly on receptor activation rather than on receptor phosphorylation.. J Biol Chem.

[15] Zhou H, Ranish JA, Watts JD, Aebersold R (2002). Quantitative proteome analysis by solid-phase isotope tagging and mass spectrometry.. Nat Biotechnol.

[16] Sklan EH, Podoly E, Soreq H (2006). RACK1 has the nerve to act: structure meets function in the nervous system.. Prog Neurobiol.

[17] Parent A, Laroche G, Hamelin E, Parent JL (2008). RACK1 regulates the cell surface expression of the G protein-coupled receptor for thromboxane A(2).. Traffic.

[18] Wang W, Huang Y, Zhou Z, Tang R, Zhao W (2002). Identification and characterization of AGTRAP, a human homolog of murine Angiotensin II Receptor-Associated Protein (Agtrap).. Int J Biochem Cell Biol.

[19] Vogler O, Bogatkewitsch GS, Wriske C, Krummenerl P, Jakobs KH (1998). Receptor subtype-specific regulation of muscarinic acetylcholine receptor sequestration by dynamin. Distinct sequestration of m2 receptors.. J Biol Chem.

[20] Jones KT, Echeverry M, Mosser VA, Gates A, Jackson DA (2006). Agonist mediated internalization of M2 mAChR is beta-arrestin-dependent.. J Mol Signal.

[21] Bolger GB, Baillie GS, Li X, Lynch MJ, Herzyk P (2006). Scanning peptide array analyses identify overlapping binding sites for the signalling scaffold proteins, beta-arrestin and RACK1, in cAMP-specific phosphodiesterase PDE4D5.. Biochem J.

[22] Pals-Rylaarsdam R, Hosey MM (1997). Two homologous phosphorylation domains differentially contribute to desensitization and internalization of the m2 muscarinic acetylcholine receptor.. J Biol Chem.

[23] Yaka R, Thornton C, Vagts AJ, Phamluong K, Bonci A (2002). NMDA receptor function is regulated by the inhibitory scaffolding protein, RACK1.. Proc Natl Acad Sci U S A.

[24] Ai E, Skop AR (2009). Endosomal recycling regulation during cytokinesis.. Commun Integr Biol.

[25] Nikolov EN, Ivanova-Nikolova TT (2004). Coordination of membrane excitability through a GIRK1 signaling complex in the atria.. J Biol Chem.

[26] Ron D, Chen CH, Caldwell J, Jamieson L, Orr E (1994). Cloning of an intracellular receptor for protein kinase C: a homolog of the beta subunit of G proteins.. Proc Natl Acad Sci U S A.

[27] Dell EJ, Connor J, Chen S, Stebbins EG, Skiba NP (2002). The betagamma subunit of heterotrimeric G proteins interacts with RACK1 and two other WD repeat proteins.. J Biol Chem.

[28] Yaka R, Phamluong K, Ron D (2003). Scaffolding of Fyn kinase to the NMDA receptor determines brain region sensitivity to ethanol.. J Neurosci.

[29] Mamidipudi V, Zhang J, Lee KC, Cartwright CA (2004). RACK1 regulates G1/S progression by suppressing Src kinase activity.. Mol Cell Biol.

[30] Sambrook J, Fritsch EF, Maniatis T (1989). Molecular Cloning: A Laboratory Manuel..

[31] Yi EC, Lee H, Aebersold R, Goodlett DR (2003). A microcapillary trap cartridge-microcapillary high-performance liquid chromatography electrospray ionization emitter device capable of peptide tandem mass spectrometry at the attomole level on an ion trap mass spectrometer with automated routine operation.. Rapid Commun Mass Spectrom.

[32] Keller A, Nesvizhskii AI, Kolker E, Aebersold R (2002). Empirical statistical model to estimate the accuracy of peptide identifications made by MS/MS and database search.. Anal Chem.

[33] Schlador ML, Grubbs RD, Nathanson NM (2000). Multiple topological domains mediate subtype-specific internalization of the M2 muscarinic acetylcholine receptor.. J Biol Chem.

[34] Goldman PS, Nathanson NM (1994). Differential role of the carboxyl-terminal tyrosine in down-regulation and sequestration of the m2 muscarinic acetylcholine receptor.. J Biol Chem.

